# Investigation of Pulmonary Saddle Embolism Caused by Metastasis-Induced Hypercoagulability Originating From Pancreatic Cancer

**DOI:** 10.7759/cureus.63477

**Published:** 2024-06-29

**Authors:** Esther Park, Milenko T Petrovic, Alexa N Pearce, Mary A Phillips, Jeanette Ramos

**Affiliations:** 1 Pathology, University of Arkansas for Medical Sciences, Little Rock, USA

**Keywords:** venous thromboembolism, pulmonary failure, pancreas cancer metastases, saddle pulmonary embolism, malignancy-associated hypercoagulability, pancreatic malignancy

## Abstract

An adult male cadaver, approximately 60 years of age, was dissected as part of an eight-week didactic course. It was found that the subject had evidence of pancreatic cancer with signs of metastasis as well as significant bilateral pulmonary artery clotting. In particular, a saddle embolism was observed, and the cause of death was listed as sudden pulmonary failure. Malignant tumors are often accompanied by hypercoagulable states and increased risk of thromboembolism. Because the clots showed lines of Zahn on histology, we can infer that this hypercoagulable state preceded death and may have been related to the presence of pancreatic carcinoma. There are few recorded cases of pulmonary saddle embolism being the fatal event in cases of underlying pancreatic cancer. The extensive clotting observed in the inferior vena cava and pulmonary arteries demonstrates to clinicians that patients, especially those with pancreatic cancer, are at higher risk for thromboembolic events. This case report also serves as a reminder that instances of pulmonary failure or sudden death because of pulmonary saddle embolism may be caused by underlying visceral neoplasms, such as pancreatic cancer.

## Introduction

Cancer diagnosis and treatment have been found to drastically increase the incidence of venous thromboembolism (VTE), leading to increased morbidity and mortality [1]. Indeed, postmortem assessments have demonstrated that VTE may occur in as many as 50% of cancer patients. Patients with pancreatic cancer may be at heightened risk for VTE. As of 2019, pancreatic cancer was the fourth-leading cause of cancer-related deaths in the United States, with VTE affecting approximately 40% of pancreatic cancer patients throughout their illness [2-4]. When clots break from venous walls and travel through the heart and pulmonary arteries, VTE leads to pulmonary embolism (PE), which can result in sudden death. While cancer patients have been found to experience a 2.87% incidence of PE during a follow-up period of six years, pancreatic cancer patients in particular have been reported to have a PE incidence of up to 5.81% during the same period [5]. Because of the insidious course of pancreatic cancer development, individuals with PE may die of PE before receiving this cancer diagnosis. Our study aimed to assess the specific link between pancreatic cancer and large-scale pulmonary emboli resulting in respiratory failure and sudden death.

## Case presentation

Deep dissection was performed throughout the body to determine anatomical variations and pathologies. The heart was weighed, and the myocardium surrounding the right and left ventricles was measured. Biopsies were taken from enlarged lymph nodes located anterosuperior to the trachea and posterior to the portal triad. Biopsies were also taken from the clotted blood in the lung vasculature, the lung tissue, the pancreas, and the liver.

**Figure 1:**
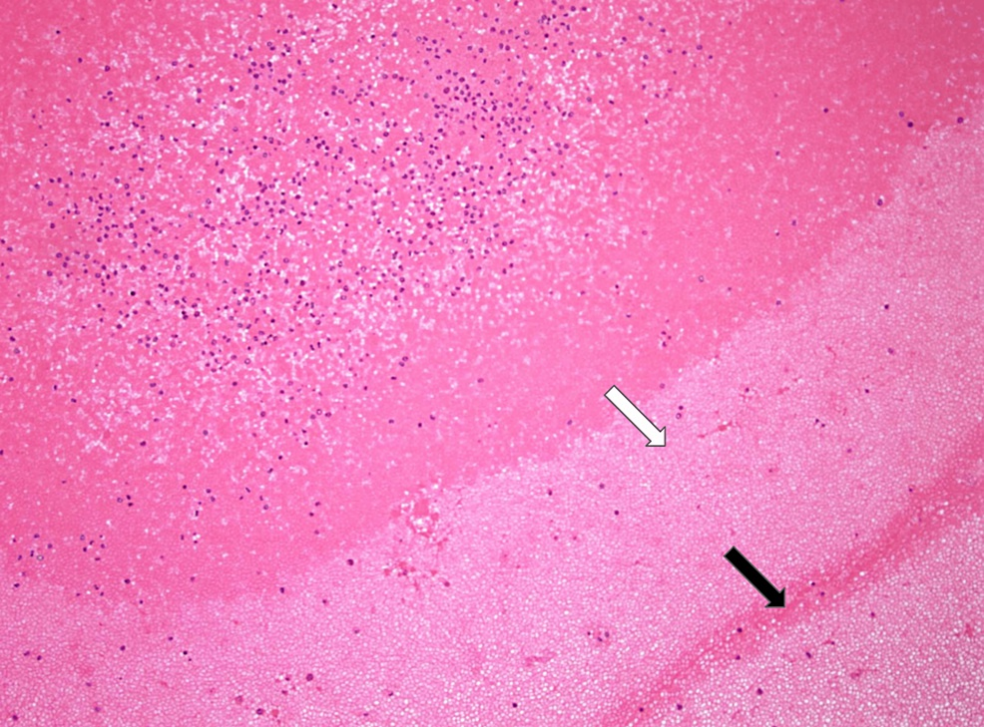
Shown are lines of Zahn, which are characteristic of thrombus formation. Platelet and fibrin layers (white arrow) alternate with red blood cell layers (black arrow).

**Figure 2:**
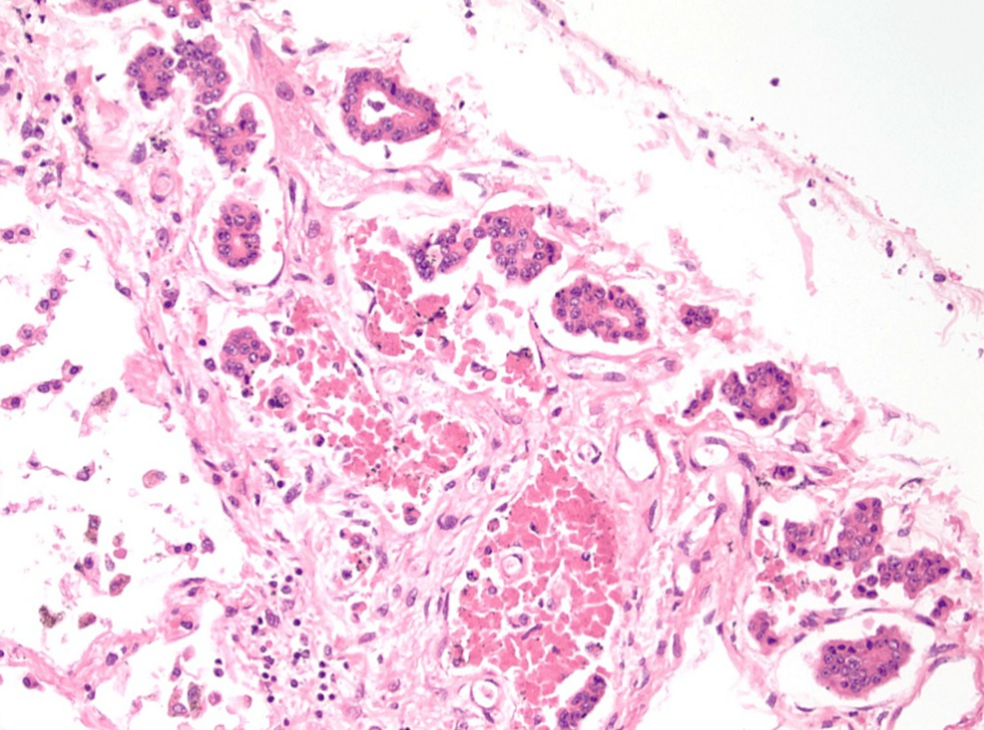
Shown are lung metastases.

**Figure 3:**
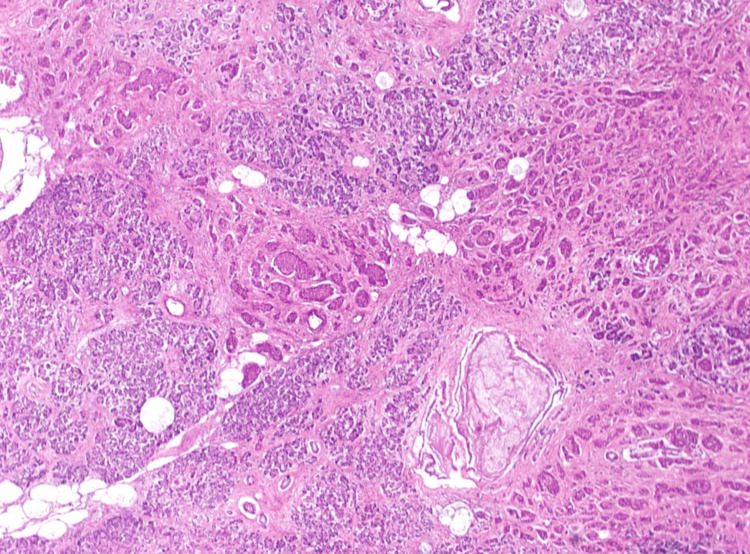
Pancreas cross-section image shows extensive autolysis. The pancreas may have been the source of the metastasized carcinoma as evidenced by scant cytoplasm and hyperchromatic nuclei.

**Figure 4:**
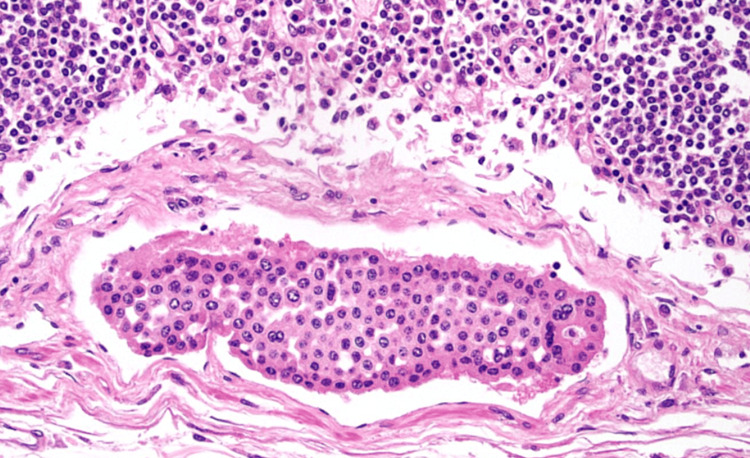
Shown are lymph node metastases. Abundant histiocytes are visible.

The cadaveric subject was approximately 220 pounds. The heart measured at 1 lb and 4.38 oz, with right and left ventricles approximately 5 and 10 mm, respectively. There was no evidence of surgical history and no detectable bruising. Gross examination revealed moderate fluid accumulation and no signs of trauma in the abdominal and thoracic cavities. However, there were signs of cardiomegaly in both ventricles of the heart, along with significant distention and clotting of the inferior vena cava. Our cadaver also showed signs of hyaloserositis (sugar-coating) in the spleen. Upon deeper dissection of the pulmonary arteries and inferior vena cava, significant and dark-colored clotting was observed. A sample of the clotted blood within the pulmonary artery showed lines of Zahn, which is a telltale sign that these clots existed before death (Figure 1). A sample taken from the pancreas also showed signs of carcinoma, as seen in Figure 2, along with severe autolysis as seen in Figure 3. Signs of metastatic carcinoma were found in both the tissue of the lung and lymph nodes (Figure 4).

## Discussion

Tissues were fixed in formalin and embedded in paraffin, allowing for 4-5-μm-thick sectioning using a microtome. Sections were heated in a pH 6.0 citrate buffer solution from Thermo Fisher Scientific for antigen retrieval. Primary antibodies of CK7 and CK20 were diluted in phosphate-buffered saline (pH 7.4) with Tween 20 (Thermo Fisher Scientific, Waltham, MA, USA). Secondary antibody incubation was performed and visualized using 3,3’ 98+% diaminobenzidine from Thermo Fisher Scientific. CK7 and CK20 antibodies were purchased through Abcam.

The biopsy showed significant autolysis in the pancreas, liver, and spleen. The stomach showed no signs of carcinoma and minor autolysis. The heart was fibrotic but not remarkably so. There was evidence of shock liver from reduced blood flow; however, it was too severely necrosed to fully determine. The pancreas was the most necrosed out of all the organs observed. The samples stained positive for CK7 and negative for CK20, consistent with the staining profile of carcinomas of the pancreas [6].

Limitations included the inability to biopsy every potential source of metastasis to rule out other carcinomas. For example, carcinomas of the extrahepatic biliary tract also tend to be CK7+ and CK20+ [6]. Additionally, studies have shown that venous thrombosis risk is correlated with the location of the tumor within the pancreas. Areas such as the corpus and cauda are associated with higher coagulability, and we could not further investigate this structural relationship in our cadaver’s pancreas because of its heavily necrosed state [7].

As mentioned before, most visceral carcinomas, such as pancreatic, lung, prostate, and colon cancer, have been consistently shown to increase the risk of venous thrombosis. However, there is currently not much literature discussing the connection between specifically pancreatic cancer and PEs, although pancreatic cancer has been known to cause a greater incidence of thromboembolic events. In this case, multiple metastases were observed in the lungs and lymph nodes of the cadaver, and instances of metastasis have been shown to increase the risk of venous thrombosis [8] greatly.

Future directions may include investigation into other malignancies commonly causing hypercoagulable states, as well as increased clinical emphasis on VTE prevention and screening of patients with malignant tumors.

## Conclusions

While the donor’s official cause of death was listed as pulmonary failure, these findings have led us to refine the cause of death to conclude that it was a saddle embolism stemming from hypercoagulability caused by pancreatic adenocarcinoma. This is supported by the extent of autolysis in the pancreas alongside the lung and lymph node metastases. Moreover, we observed distension and clotting present in the inferior vena cava. As stated before, patients with pancreatic cancer, in particular, are at risk for pulmonary emboli compared to other cancer types. Finally, the donor had well-defined musculature that suggests he lived an active lifestyle, so the aggressive and sudden nature of his death shows that clinicians should pay special attention to patients with pancreatic cancer, especially concerning monitoring their coagulability to prevent catastrophic events related to major embolisms.
